# MsmiR171 targets *MsSCL6* to mediate selenium-regulated chlorophyll biosynthesis in alfalfa

**DOI:** 10.1093/hr/uhaf305

**Published:** 2025-11-13

**Authors:** Qingdong Wang, Shuting Su, Yarui Sheng, Mengli Xu, Baohong Tang, Yonggui Ma, Yuhua Shi

**Affiliations:** School of Life Sciences, Zhengzhou University, Zhengzhou 450001, Henan, China; Henan Key Laboratory of Bioactive Macromolecules, Zhengzhou 450001, Henan, China; Key Laboratory of Medicinal Animal and Plant Resources of Qinghai Tibetan Plateau, Qinghai Normal University, Xining 810008, China; School of Life Sciences, Zhengzhou University, Zhengzhou 450001, Henan, China; Henan Key Laboratory of Bioactive Macromolecules, Zhengzhou 450001, Henan, China; Key Laboratory of Medicinal Animal and Plant Resources of Qinghai Tibetan Plateau, Qinghai Normal University, Xining 810008, China; School of Life Sciences, Zhengzhou University, Zhengzhou 450001, Henan, China; Henan Key Laboratory of Bioactive Macromolecules, Zhengzhou 450001, Henan, China; Key Laboratory of Medicinal Animal and Plant Resources of Qinghai Tibetan Plateau, Qinghai Normal University, Xining 810008, China; School of Life Sciences, Zhengzhou University, Zhengzhou 450001, Henan, China; Henan Key Laboratory of Bioactive Macromolecules, Zhengzhou 450001, Henan, China; Key Laboratory of Medicinal Animal and Plant Resources of Qinghai Tibetan Plateau, Qinghai Normal University, Xining 810008, China; School of Life Sciences, Zhengzhou University, Zhengzhou 450001, Henan, China; Henan Key Laboratory of Bioactive Macromolecules, Zhengzhou 450001, Henan, China; School of Life Sciences, Qinghai Normal University, Xining 810008, China; Henan Key Laboratory of Bioactive Macromolecules, Zhengzhou 450001, Henan, China; Key Laboratory of Medicinal Animal and Plant Resources of Qinghai Tibetan Plateau, Qinghai Normal University, Xining 810008, China; School of Life Sciences, Zhengzhou University, Zhengzhou 450001, Henan, China; Henan Key Laboratory of Bioactive Macromolecules, Zhengzhou 450001, Henan, China; Key Laboratory of Medicinal Animal and Plant Resources of Qinghai Tibetan Plateau, Qinghai Normal University, Xining 810008, China

## Abstract

Alfalfa (*Medicago sativa* L.) is a globally pivotal legume forage. Selenium (Se), an essential trace element for humans and animals, can significantly enhance the growth and development of alfalfa. Chlorophyll is the central pigment of plant photosynthesis. Previous research on chlorophyll synthesis in alfalfa has mainly focused on transcriptional regulation, environmental factors (light, nutrient availability), and phytohormone signaling, while fewer studies have been conducted at the post-transcriptional level. Through whole transcriptome sequencing analysis, microRNAs (miRNAs) were identified as positively responsive to Se. This study focused on the regulation of chlorophyll synthesis by the miR171-*SCL6* module in alfalfa. β-glucuronidase staining and dual-luciferase assays revealed that MsmiR171 negatively regulated the transcript levels of the *SCARECROW-LIKE 6* transcription factor *MsSCL6.* Subcellular localization analysis revealed that MsSCL6 was mainly in the cell nucleus. Functional analyses demonstrated that MsmiR171 promoted chlorophyll synthesis and photosynthesis in alfalfa, while *MsSCL6* negatively regulated chlorophyll synthesis. Notably, Se treatment upregulated MsmiR171 expression, downregulated *MsSCL6* expression*,* and enhanced chlorophyll accumulation. qRT-PCR analysis revealed differential expression of *MsPOR* in MsmiR171 and *MsSCL6* overexpression or silencing plants. Combined yeast one-hybrid and dual-luciferase assays demonstrated that MsSCL6 transcriptionally represses *MsPOR* through direct promoter binding, suppressing chlorophyll accumulation. In summary, this study for the first time revealed the mechanism of the MsmiR171-*MsSCL6*-*MsPOR* module mediating Se-regulated chlorophyll biosynthesis in alfalfa. These findings provide a theoretical foundation and technical guidance for alfalfa breeding and the production of Se-enriched forage.

## Introduction

Selenium (Se) is an essential trace element that humans and animals cannot endogenously synthesize, and its intake primarily occurs through the consumption of plant-based foods [1]. Epidemiological evidence demonstrates a significant association between chronic Se inadequacy and endemic pathologies, including Keshan disease and Kashin–Beck disease [2]. Therefore, biofortification of Se, particularly through agricultural techniques to enhance the levels of micronutrients in crops, is one of the crucial strategies to prevent Se deficiency in humans and animals [3]. Se functions dually as a required micronutrient and possible toxin [4]. Low-dose Se enhances plant growth, development, and yield [5]. Presently, Se’s effects on plant antioxidant capacity and photosynthesis are one of the research hotspots. For instance, the application of Se enhances photosynthetic efficiency and antioxidant capacity in *Raphanus sativus*, concomitant with elevated nutritional components, including soluble sugars, soluble proteins, and anthocyanins [6]. Exogenous Se application also improves photosynthetic rates and photosystem II (PSII) activity in economically important species, such as rice and *Stevia rebaudiana* [7, 8]. These findings collectively advance our understanding of Se-mediated metabolic regulation in plants.

MicroRNA (miRNA) is a class of short noncoding RNAs (20–24 nt) that regulate gene expression at the posttranscriptional level through mRNA cleavage or translational inhibition, thereby controlling core biological functions such as plant growth, development, and stress adaptation [9, 10]. Extensive interaction networks between miRNAs and transcription factors (TFs) have been well characterized in recent studies [11]. For instance, the miR319 modulates leaf morphogenesis by suppressing TCP family TFs [12]. The miR165/16*6-PHABULOSA* (*PHB*) module controls heat stress responses via HSFA1 regulation [13]. miR166e-*ZmATHB14* module regulates drought tolerance in the maize root [14]. The regulatory module consisting of miR164a and *NAM3* improves tomato cold resistance through the activation of ethylene biosynthesis pathways [15]. miR171 represents a conserved miRNA family [16]. Studies have shown that the miR171 targets the *HAM* gene (*Scarecrow-like 6*, *SCL6*). This gene produces a GRAS family transcription factor, specific to plants, which is composed of GAI, RGA, and SCR homologs, and is one of the largest TF families in plants [17]. Furthermore, studies have shown that the miR171-*SCL6* module can participate in branch formation [18], meristem maintenance [19], root growth [20], and somatic embryogenesis (SE) processes [21], as well as plant drought and verticillium wilt resistance regulation [22, 23]. In addition, miR171 is associated with *SCL* in regulating plant chlorophyll content. Overexpression of miR171 in *Arabidopsis* and triple *scl6* mutants can affect various phenotypes such as plant height, chlorophyll accumulation, flower structure and leaf shape [18], and its module can cooperate with the GA-DELLA pathway to regulate chlorophyll biosynthesis under light [24]. Similarly, the bol-miR171b-*BolSCL6* module in broccoli has also been shown to regulate chlorophyll content [25]. As the primary pigment in photosynthesis, chlorophyll plays a critical role in enhancing the potential photosynthetic rate and, to some extent, increasing plant biomass [26]. In *Arabidopsis*, researchers have identified a complex chlorophyll biosynthesis pathway. This process involves a series of 15 distinct enzymes, coded by 27 different genes [27]. Multiple factors regulate chlorophyll accumulation, such as the structural integrity of the chloroplast, external conditions, plant hormonal regulation, and transcriptional control of essential chlorophyll biosynthesis genes. Studies have revealed that the miR171-*SCL* module might govern chlorophyll biosynthesis by modulating critical genes responsible for chlorophyll biosynthesis, which in turn impacts photosynthetic efficiency and biomass growth. These results provide novel perspectives on the molecular mechanisms of miRNA-TF regulatory networks in the process of photosynthesis. However, the role of the miR171-*SCL* module in the chlorophyll synthesis of alfalfa has yet to be revealed.

Alfalfa (*Medicago sativa* L.) is a perennial legume forage, and its yield and quality are crucial for ensuring global feed supply [28]. Alfalfa is not only rich in protein, digestible fiber, vitamins, and minerals, but also contains bioactive compounds such as saponins, polysaccharides, and flavonoids, earning it the title of the ‘king of forage’ [29]. However, some factors, including unfavorable environmental conditions (drought, low temperature, and soil salinity), limited availability of improved varieties, and suboptimal field management practices, often result in yields and quality that fail to meet the demands of animal husbandry [30]. Therefore, clarifying the molecular mechanism will contribute to the production of high-quality alfalfa.

Previous studies have found that foliar Se application can significantly affect the photosynthetic rate and antioxidant capacity of alfalfa and increase forage yield [31]. Here, we further found that MsmiR171 and *MsSCL6* both respond to Se but display different expression patterns. It was noted that MsmiR171 targets *MsSCL6* and promotes chlorophyll biosynthesis. Meanwhile, MsSCL6 inhibited the expression of key enzyme genes in the chlorophyll synthesis pathway. This result suggests that the MsmiR171-*MsSCL6* module regulates chlorophyll biosynthesis by mediating Se in alfalfa, providing a molecular strategy to enhance photosynthetic efficiency and breed Se-enriched forage crops.

## Results

### Bioinformatics analysis

In the previous whole transcriptome analysis, we observed Se-induced upregulation of MsmiR171 expression. The precursor sequence of MsmiR171 was predicted to have a steady hairpin structure by the RNAfold web (Fig. S1A; Table S1). Analysis of sequence alignment revealed that the mature sequence of MsmiR171 shares similarity with miR171 sequences found in *Arabidopsis* (Fig. S1B). The primary mode of action of miRNAs has shown that miR171 targets *SCLs*, modulating plant developmental processes [19, 32]. As a result, *MsSCL6* (*MS.gene48898.t1*), which may be a target gene of MsmiR171, was identified through the psRNATarget program (Fig. S1C). qRT-PCR analysis showed significant up-regulation of MsmiR171 and down-regulation of *MsSCL6* under Se application (Fig. S1D and E). These results imply that both MsmiR171 and *MsSCL6* might participate in the Se response, and their expression trends showed a negative correlation, providing indirect evidence that *MsSCL6* is a target gene of MsmiR171. Additionally, bioinformatics analysis of MsSCL6 was performed to facilitate follow-up experimental studies. The CDS of *MsSCL6* contains 1884 bp, which encodes a 627 amino acid protein (Table S2). The results of domain analysis showed that MsSCL6 had a GRAS conserved domain (Fig. S1G). A phylogenetic analysis also revealed that MsSCL6 and AtSCL6 were clustered into the same HAM subfamily (Fig. S1F; Table S3).

### Subcellular localization

**Figure 1:**
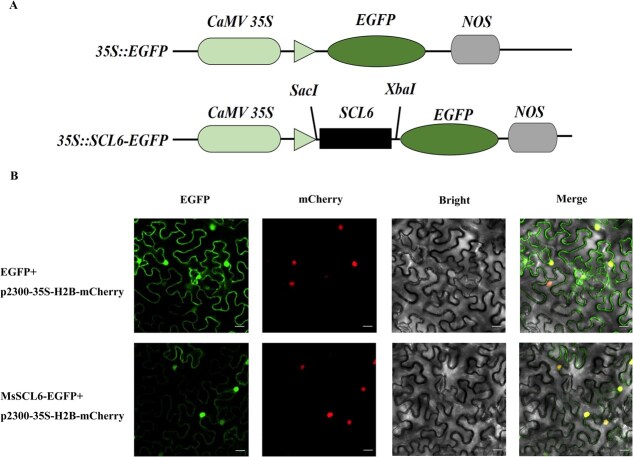
Subcellular localization of MsSCL6. **A** vector construction schematic diagram. **B** MsSCL6 subcellular localization in *N. benthamiana* leaves, with a scale bar of 22 μm. p2300-35S-H2B-mCherry serves as a reference vector for nuclear localization. The location of the nucleus can be determined by observing the fluorescence of mCherry.

To analyze MsSCL6 subcellular localization, the MsSCL6-EGFP vector was constructed and introduced into tobacco leaves (Fig. 1A). The control group utilized an EGFP empty vector. Confocal microscopy revealed that the fluorescence signal of the MsSCL6-EGFP fusion protein was predominantly localized in the nucleus. (Fig. 1B). Nuclear localization was predicted for the protein by subcellular localization analysis (Fig. S2). Our findings indicate that the MsSCL6 protein is primarily nuclear-localized, which aligns with its predicted function as a transcription factor. However, the observed fluorescence signals at the cell membrane require further validation through additional experiments, such as colocalization assays.

### *MsSCL6* is the target gene of MsmiR171

**Figure 2:**
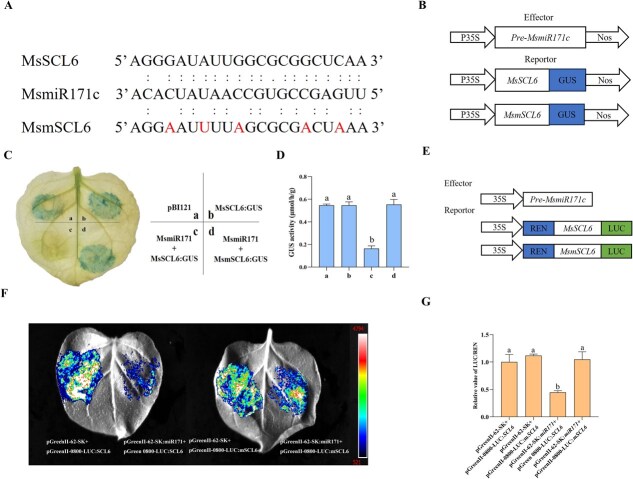
MsmiR171 inhibits the expression of *MsSCL6*. **A** Schematic illustration of the mutation within the MsmiR171 target site. The nucleotides at positions 4, 7, 10, 16, and 19 of MsmSCL6 are mutated bases. **B** Effector and reporter vectors construction. **C, D** GUS histochemical staining and activity quantification at agroinfiltrated leaf sites containing different vectors. pBI121(35S: GUS). **E** Effector and reporter vectors construction. **F, G** Luciferase imaging assay and relative luciferase activity assay. The ratio of luciferase: Renilla (LUC/REN) for the combination of the pGreenII-62-SK vector and the pGreenII 0800-*MsSCL6* vector was normalized to 1. Different colors mean luciferase activity. The data is shown as mean ± SD (*n* = 3), with different letters signifying statistically significant differences at *P* < 0.05.

To demonstrate whether MsmiR171 directly targets *MsSCL6* or not, the expression levels of the *MsSCL6* gene were quantified. The expression of *MsSCL6* was in contrast to MsmiR171 (Fig. S1D and E). Histochemical staining showed a similar GUS phenotype in leaves infiltrated with pBI121 and *MsSCL6*-GUS, and the leaves of cotransformed MsmiR171 and *MsmSCL6*-GUS, but cotransformed MsmiR171 and *MsSCL6*-GUS significantly reduced staining intensity (Fig. 2A–C). To verify these histochemical staining results, GUS activity in *Nicotiana benthamiana* leaves was measured. The measurements were consistent with the phenotypic observations (Fig. 2D). Additionally, to better understand the interaction between MsmiR171 and *MsSCL6,* a dual-luciferase assay was tested. MsmiR171 was co-expressed in *N. benthamiana* leaves with *MsmSCL6,* and strong fluorescence signals were observed. However, co-expression of MsmiR171 with *MsSCL6* resulted in a significant decrease in fluorescence intensity (Fig. 2E and F). The LUC/REN values were consistent with the fluorescence intensity (Fig. 2G). These results demonstrated that MsmiR171 negatively regulates *MsSCL6* expression.

### MsmiR171 positively regulates chlorophyll accumulation and photosynthesis under Se application

**Figure 3:**
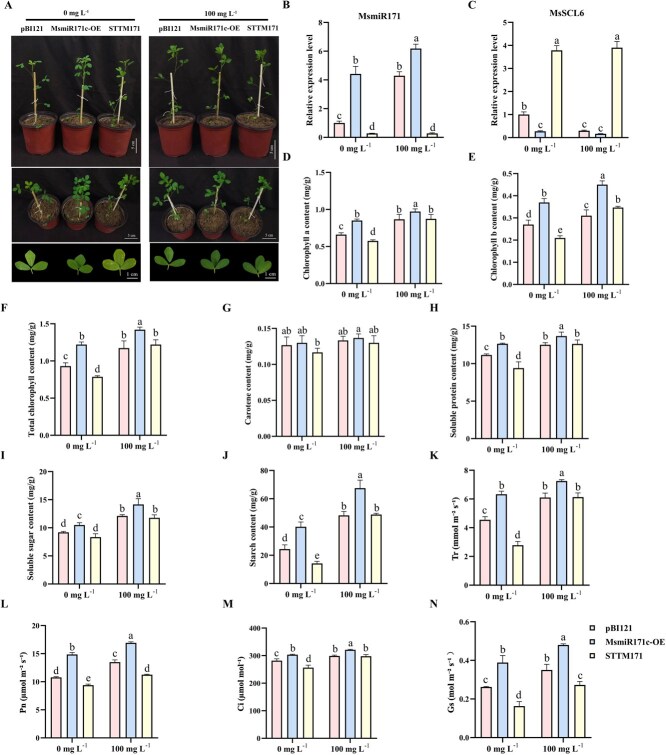
MsmiR171 positively regulates chlorophyll synthesis in alfalfa. **A** Effects of Se application on the phenotypes of control, MsmiR171-OE, and STTM171 plants. **B** Effects of Se application on MsmiR171 expression in control, MsmiR171-OE, and STTM171 plants. **C** Effects of Se application on *MsSCL6* expression in control, MsmiR171-OE, and STTM171 plants. Contents of **D** Chlorophyll a, **E** Chlorophyll b, **F** Total chlorophyll, **G** Carotenoids, **H** Soluble protein, **I** Soluble sugar, and **J** Starch, **K** Tr, **L** Pn, **M** Ci, and **N** Gs in control, MsmiR171-OE, and STTM171 plants before and after Se application. The pBI121 served as the control. The data is shown as mean ± SD (*n* = 3), with different letters signifying statistically significant differences at *P* < 0.05.

To determine the function of MsmiR171 in alfalfa, we generated MsmiR171-OE and STTM171 constructs. The expression of MsmiR171 was significantly increased in the MsmiR171-OE plants and decreased in the STTM171 plants. Under Se application or without application, MsmiR171 overexpression inhibited *MsSCL6* expression, whereas STTM171 plants increased *MsSCL6* expression (Fig. 3B and C). These results confirmed the existence of a miR171-*MsSCL6* regulatory cascade during the growth and development of alfalfa.

Moreover, MsmiR171-OE plants exhibited darker leaf coloration than the control, with increased chlorophyll, soluble protein, soluble sugar, and starch levels, while no significant difference was shown in carotenoid content. In contrast, the STTM171 plants exhibited leaf chlorosis, with significantly reduced levels of chlorophyll, soluble protein, and starch, while soluble sugar and carotenoid levels showed no significant variation. Interestingly, all plants displayed a deepening of leaf color and a further increase in chlorophyll, soluble protein, soluble sugar and starch levels following Se application, while carotenoid content had no significant effect. Meanwhile, the chlorosis in the STTM171 plants was alleviated (Fig. 3A and D-J). These results demonstrated that MsmiR171 positively regulates chlorophyll synthesis and quality traits in alfalfa.

To investigate the impact of MsmiR171 on photosynthetic ability, we assessed the photosynthetic performance of alfalfa leaves. The MsmiR171-OE plants showed significantly enhanced Tr, Pn, Ci, and Gs compared to control plants. Contrarily, the STTM171 plants exhibited marked reductions in Tr, Pn, Ci, and Gs. After Se application, all plants maintained significantly higher photosynthetic parameters (Fig. 3K–N). These results indicated that MsmiR171 can influence photosynthesis in alfalfa leaves by altering chlorophyll content, and Se application enhances photosynthetic activity in the leaves of different plants, thereby affecting plant biomass accumulation.

### Effects of *MsSCL6* overexpression on chlorophyll accumulation and photosynthesis under Se application

**Figure 4:**
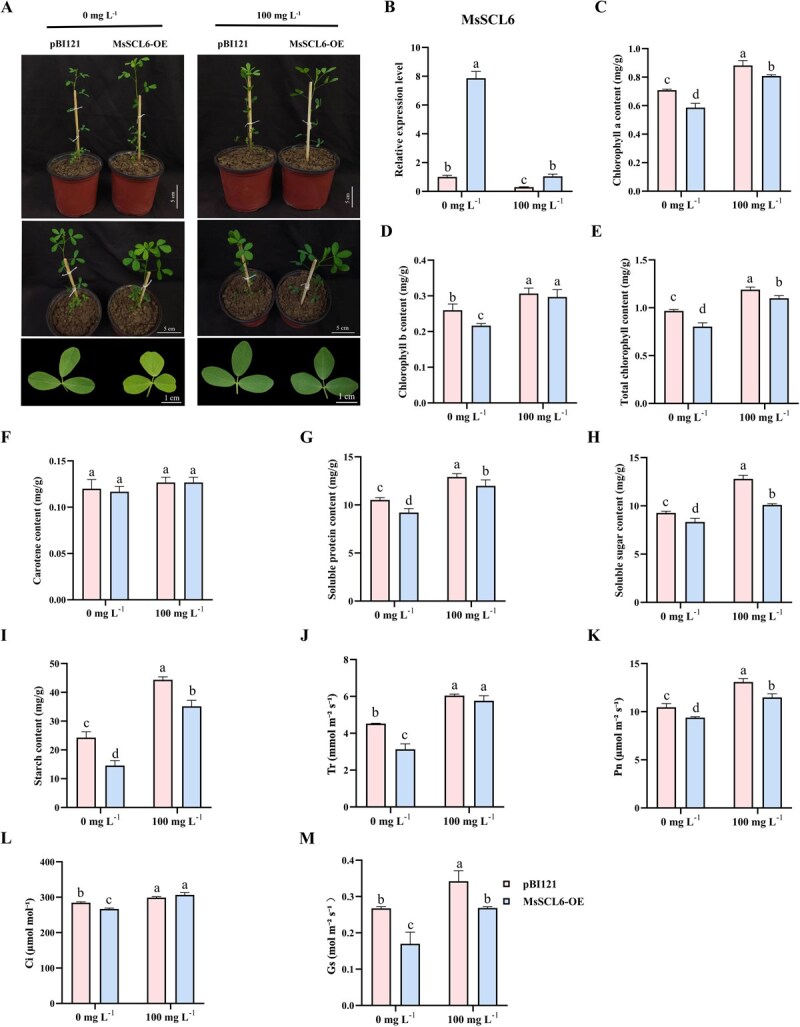
The effect of *MsSCL6*-OE on chlorophyll synthesis in alfalfa. **A** Effects of Se application on the phenotypes of control and *MsSCL6*-OE plants. **B** Effects of Se application on *MsSCL6* expression in control and *MsSCL6*-OE plants. Contents of **C** Chlorophyll a, **D** Chlorophyll b, **E** Total chlorophyll, **F** Carotenoids, **G** Soluble protein, **H** Soluble sugar, and **I** Starch, **J** Tr, **K** Pn, **L** Ci, and **M** Gs in control and *MsSCL6*-OE plants before and after Se application. The pBI121 served as the control. The data is shown as mean ± SD (*n* = 3), with different letters signifying statistically significant differences at *P* < 0.05.

To characterize *MsSCL6*’s role in chlorophyll biosynthesis, the *MsSCL6* overexpression vector (*MsSCL6*-OE) was constructed. Compared with the control, a substantial increase in *MsSCL6* transcript levels was observed in the *MsSCL6*-OE plants, indicating successful overexpression. qRT-PCR analysis showed that *MsSCL6* expression in *MsSCL6*-OE plants did not differ significantly from the untreated controls under Se application (Fig. 4B). Using the *MsSCL6*-OE plants as experimental material, the effect of *MsSCL6* expression level on chlorophyll content and quality traits in alfalfa was observed. The *MsSCL6*-OE plants showed that the color of their leaves turned yellow compared with the control, along with significantly lower levels of chlorophyll, soluble protein, soluble sugar, and starch. After Se application, the leaf color of both the control and *MsSCL6*-OE plants darkened, and the chlorosis in the *MsSCL6*-OE plants was alleviated. The levels of chlorophyll, soluble protein, soluble sugar, and starch obviously increased in both the control and *MsSCL6*-OE plants, while carotenoid content displayed no remarkable difference (Fig. 4A and C–I). These findings indicated that *MsSCL6* negatively regulates chlorophyll biosynthesis and affects quality traits in alfalfa.

Physiological measurements further demonstrated that, before Se application, the *MsSCL6*-OE plants displayed significantly lower Tr, Pn, Ci, and Gs. After Se application, these photosynthetic parameters in the *MsSCL6*-OE plants significantly increased (Fig. 4J–M). In summary, *MsSCL6* negatively regulates chlorophyll synthesis and photosynthesis, while Se application alleviates the inhibitory effects of *MsSCL6*.

### Effects of *MsSCL6* silencing on chlorophyll accumulation and photosynthesis under Se application

**Figure 5:**
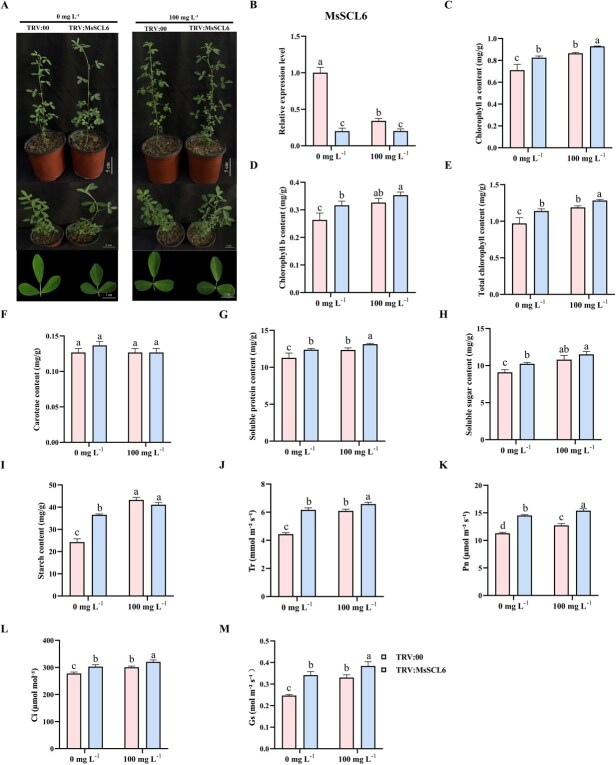
The effect of TRV: *MsSCL6* on chlorophyll synthesis in alfalfa. **A** Effects of Se application on the phenotypes of control and TRV: *MsSCL6* plants. **B** Effects of Se application on *MsSCL6* expression in control and TRV: *MsSCL6* plants. Contents of **C** Chlorophyll a, **D** Chlorophyll b, **E** Total chlorophyll, **F** Carotenoids, **G** Soluble protein, **H** Soluble sugar, and **I** Starch, **J** Tr, **K** Pn, **L** Ci, and **M** Gs in control and TRV: *MsSCL6* plants before and after Se application. The TRV: 00 served as the control. The data is shown as mean ± SD (*n* = 3), with different letters signifying statistically significant differences at *P* < 0.05.

To further verify the function of *MsSCL6* in chlorophyll synthesis in alfalfa, we generated TRV: *MsSCL6*-silenced plants by virus-induced gene silencing (VIGS). qPCR analysis revealed that, before Se application, the expression of *MsSCL6* in TRV: *MsSCL6* plants was notably reduced in comparison with the control. After Se application, the expression of *MsSCL6* in TRV: *MsSCL6* plants showed no significant difference compared to that before Se treatment, and it was still lower than that of the untreated control (Fig. 5B). These results indicated that Se can suppress the expression of *MsSCL6*. Additionally, we observed that inhibiting the *MsSCL6* gene resulted in darker leaf coloration and a marked increase in levels of chlorophyll, soluble protein, soluble sugar, and starch, while there was no notable change in carotenoid levels. After Se application, the leaf color of both the control and TRV: *MsSCL6* plants became darker, and there was an obvious increase in the levels of chlorophyll, soluble protein, soluble sugar, and starch (Fig. 5A and C–I). These results suggested that *MsSCL6* negatively regulates chlorophyll synthesis and affects plant growth and development under the application of Se.

In this study, we also analyzed photosynthetic capacity in TRV: *MsSCL6* plants and control plants. Before Se application, the TRV: *MsSCL6* plants exhibited significantly higher Tr, Pn, Ci, and Gs than the control plants. After Se application, the photosynthetic capacity of all plants improved, showing a significant difference between the silenced and the control plants. This indicated that TRV: *MsSCL6* plants had higher photosynthetic capacity, and Se could enhance the photosynthesis of different lines (Fig. 5J–M).

### MsmiR171 and *MsSCL6* regulate chloroplast development

**Figure 6:**
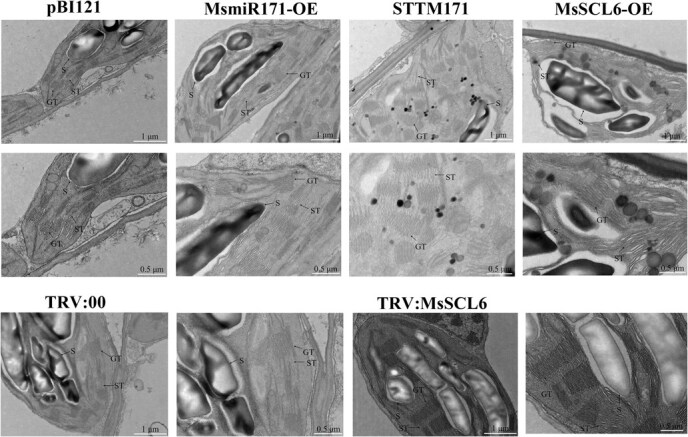
The chloroplast ultrastructure in the leaves of MsmiR171 and *MsSCL6* overexpressed and silenced plants was observed by transmission electron microscopy (TEM). S: Starch granule; GT: Granum thylakoid; ST: Stroma thylakoid.

The chloroplast ultrastructure of MsmiR171 and *MsSCL6* gene expression-regulated plants was analyzed by transmission electron microscopy. The results showed that the overall chloroplast structure of MsmiR171-OE plants was the same as that of the empty control pBI121, the chloroplast membrane system was complete, the grana lamellae were arranged in order, and the volume of starch granules exhibited a progressive increasing trend. However, compared with the control, MsmiR171-OE plants showed a more complex thylakoid membrane system, and an increasing trend in the number of grana stacking layers and thylakoid membrane layers was observed. In contrast, the chloroplasts of STTM171 plants showed significant abnormalities, manifested as membrane structure damage, local thylakoid lamellar dissolution, and starch granules exhibited a tendency toward reduced volume and vacuolation. In the *MsSCL6* gene regulation system, the chloroplast development of *MsSCL6*-OE plants was seriously hindered, which was manifested as a rough surface of membrane structure, disordered arrangement, and a reducing trend in the number of thylakoid lamellae, and large-scale vacuolization around starch granules. Compared with TRV:00, although there was no significant difference in chloroplast morphology, size, and starch granule charact4eristics between TRV: *MsSCL6* gene silencing plants and empty control TRV:00, the number of thylakoid membrane layers of grana exhibited an upward tendency (Fig. 6). This indicated that MsmiR171 and *MsSCL6* may be involved in regulating chloroplast development.

### Regulation of chlorophyll biosynthesis by the MsmiR171-*MsSCL6* module through modulating the expression of key genes in the chlorophyll synthesis pathway under Se application

To further investigate the role of the MsmiR171-*MsSCL6* module in chlorophyll synthesis in alfalfa, we analyzed the expression levels of 10 chlorophyll-synthesis-related genes between control and treated plants. We found that before Se application, the chlorophyll-related genes *MsCHLE*, *MsCAO*, *MsCHLG*, *MsPOR*, and *MsCHLD* were significantly upregulated in MsmiR171-OE plants, while *MsPOR* and *MsCHLG* showed significant downregulation in the STTM171 plants, and *MsPOR* and *MsCHLD* were significantly downregulated in the *MsSCL6*-OE plants. Under the application of Se, compared to the non-Se control, 10 chlorophyll-related genes showed markedly elevated expression in MsmiR171-OE plants. *MsCHLE*, *MsDVR*, *MsCAO*, *MsCHLG*, *MsPOR*, *MsCHLI*, and *MsCHLD* were significantly upregulated in *MsSCL6*-OE plants, while *MsHEMA* and *MsDVR* were significantly upregulated in STTM171 plants (Fig. S4). This indicated that Se promotes chlorophyll-related enzyme expression through the MsmiR171-*MsSCL6* module, thereby regulating chlorophyll synthesis.

We further measured chlorophyll-associated gene expression levels in TRV: *MsSCL6* plants via qRT-PCR. Compared to the control, the expression levels of *MsCAO*, *MsCHLG*, *MsPOR*, and *MsCHLD* were significantly upregulated in the TRV: *MsSCL6* plants. After Se application, the expression levels of *MsCHLE*, *MsCAO*, *MsCHLG*, *MsPOR*, *MsCHLH*, *MsCHLI*, *MsCHLD*, and *MsCHLM* in the TRV: *MsSCL6* plants were significantly upregulated relative to the non-treated control. These findings demonstrate that *MsSCL6* silencing upregulates chlorophyll-synthesis gene expression, and the application of Se can maintain or further enhance their expression in silenced plants (Fig. S5). Interestingly, the *MsPOR* gene exhibited the most significant changes across different treatment groups. Combined with literature suggesting a potential interaction between *MsSCL6* and *MsPOR*, *MsPOR* was selected for further investigation.

### MsSCL6 directly binds to the promoter of *MsPOR*

**Figure 7:**
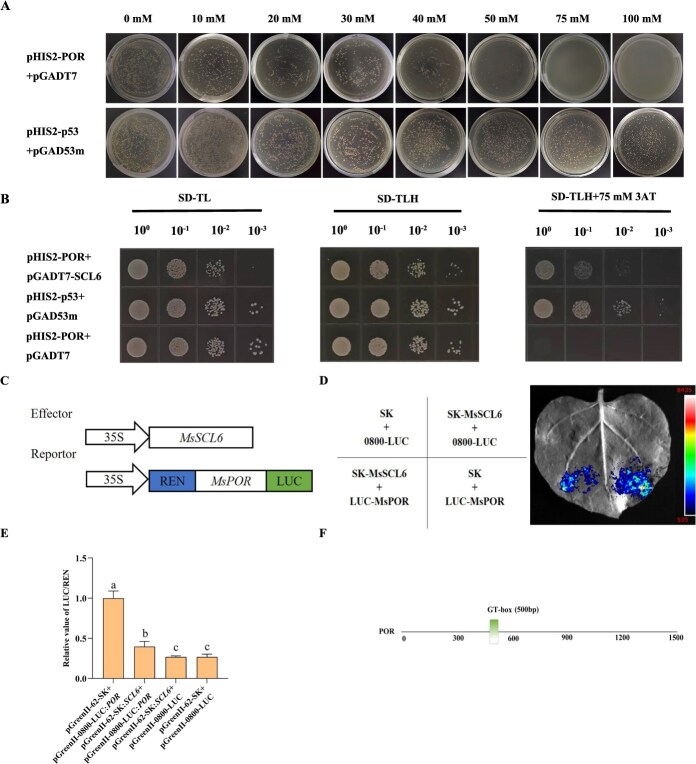
MsSCL6 negatively regulates *MsPOR* expression by binding to its promoter. **A** Growing phenotype of the Y187 yeast strain harboring pHIS2-*MsPOR* on SD/−Leu/−Trp/-His with 0–100 mM 3-AT. **B** Yeast one-hybrid (Y1H) assay shows that MsSCL6 binds to *MsPOR* promoter in *vitro*. pHIS2-p53 + pGAD53m: positive control, pHIS2-*MsPOR* + pGADT7: negative control. **C** Effector and the reporter vectors construction. **D, E** Luciferase imaging and activity assays show that MsSCL6 negatively regulates *MsPOR* expression. The ratio of luciferase: Renilla (LUC/REN) for the combination of the pGreenII-62-SK vector and the pGreenII 0800-*MsPOR* vector was normalized to 1. Different colors mean luciferase activity. The data is shown as mean ± SD (*n* = 3), with different letters signifying statistically significant differences at *P* < 0.05. **F** Promoter diagram of *MsPOR*.

Considering that the MsmiR171-*MsSCL6* module regulates chlorophyll biosynthesis in alfalfa, potentially through modulating *MsPOR* expression (*MS.gene059050.t1*), we further explored its molecular mechanism of action. Firstly, through auto-activation experiments, the Y187 yeast strain containing the pHIS2-*MsPOR* vector was cultured on SD/-Leu/-Trp/-His medium supplemented with 0–100 mM 3-AT, demonstrating that 75 mM is the optimal concentration for yeast one-hybrid (Y1H) screening (Fig. 7A). Subsequently, the Y187 yeast strain harboring both pGADT7-*MsSCL6* and pHIS2-*MsPOR* cultured on SD/-Leu/-Trp/-His containing 75 mM 3-AT. This result indicated that MsSCL6 can directly bind to the promoter region of *MsPOR* (Fig. 7B).

To further confirm MsSCL6’s regulation on *MsPOR* in alfalfa, we performed LUC reporter assays. The results indicated that cotransformed with the pGreenII-62-SK + pGreenII 0800-LUC-*MsPOR* or pGreenII-62-SK-*MsSCL6* + pGreenII 0800-LUC-*MsPOR* showed LUC fluorescence signals. The LUC fluorescence signals from the cotransfection of pGreenII-62-SK-*MsSCL6* and pGreenII 0800-LUC-*MsPOR* were attenuated. (Fig. 7C and D). Additionally, the dual-luciferase activity assay results showed that the LUC activity of pGreenII-62-SK-*MsSCL6* + pGreenII 0800-LUC-*MsPOR* had significantly decreased (Fig. 7E). These results indicated that MsSCL6 negatively regulated the expression of *MsPOR* by directly binding to its promoter. Furthermore, the promoter of *MsPOR* was predicted to contain a GT box (Fig. 7F).

## Discussion

Early studies have demonstrated that 100 mg l^−1^ Se application could deepen the color of alfalfa leaves, and increase plant chlorophyll content and photosynthetic efficiency [31]. However, the mechanism of Se-induced chlorophyll accumulation in alfalfa remains unknown. The analysis of previous whole transcriptome data and qRT-PCR verification showed that MsmiR171 expression was significantly upregulated after Se application (Fig. S1D). In some other plants, miR171 is involved in regulating diverse developmental processes, such as shoot meristem development, branch number, leaf shape, leaf color, and chlorophyll accumulation [18, 24, 32, 33]. Our current study revealed that the upregulation of MsmiR171 expression after Se application promoted chlorophyll accumulation, enhanced the photosynthesis of alfalfa, and determined its inhibitory effect on *MsSCL6*. We also found that MsSCL6 negatively regulates *MsPOR* expression by binding to its promoter. These findings establish the MsmiR171-*MsSCL6* module as a key regulator of alfalfa chlorophyll biosynthesis.

MiR171 is one of the oldest and evolutionarily conserved miRNAs in plants, with its ‘GAGCCG’ and ‘CAAUAU’ sequences representing highly conserved regions across plant species [34]. It plays pivotal roles in some plant growth and development, hormone signaling, stress responses, and microbial interactions [35–38]. Sequence alignment showed that the mature MsmiR171c differs from AtmiR171c by one nucleotide, implying functional conservation (Fig. S1B). As key post-transcriptional regulators, miR171 often acts on GRAS family genes [34]. Here, the Se application induced differential expression of MsmiR171 and *MsSCL6*, with *MsSCL6* predicted as a potential target (Fig. S1C and E). Proteins within the same family typically perform similar functions in biological processes [39]. MsSCL6 possesses a conserved GRAS domain and phylogenetically clusters with AtSCL6 (Fig. S1F). Typically, TFs regulate gene expression only in the nucleus. Our study revealed that MsSCL6 protein localizes primarily to the nucleus, consistent with its predicted TF function. However, the fluorescent signals that appear in the cell membrane require further verification through colocalization assays (Fig. 1). Post-transcriptional regulation of MsSCL6 by MsmiR171 was demonstrated through GUS staining and dual-luciferase assays, influencing downstream gene activity (Fig. 2). In general, miRNA acts on target genes through mRNA cleavage or translation inhibition [40, 41]. In this study, it was found that the mRNA level changed significantly after miR171 overexpression or silencing, suggesting that miR171 likely inhibits the expression of the target gene through mRNA cleavage.

Previous studies have reported that the miR171 family modulates chlorophyll biosynthesis and accumulation through post-transcriptional regulation of target genes in some plants. In *Arabidopsis*, it is found that the phenotype of miR171 overexpressing plants is similar to that of *scl6-II scl6-III scl6-IV* triple mutants, promoting branching and chlorophyll accumulation [18, 24]. In addition, in rice, silencing osa-miR171b caused stunting and leaf chlorosis resembling viral symptoms [32]. These findings suggest conserved roles for the MsmiR171-*MsSCL6* module in chlorophyll regulation. In this study, we showed that MsmiR171-OE and TRV: *MsSCL6* alfalfa plants exhibited darker leaf color and increased chlorophyll content (Figs 3 and 5). Conversely, STTM171 and *MsSCL6*-OE plants displayed leaf yellowing and reduced chlorophyll accumulation (Figs 3 and 4). These results were consistent with those being found in *Arabidopsis*, broccoli, and rice [18, 25, 32], which further confirmed the evolutionary conservation of this regulatory module. Interestingly, in this study, it was found that after Se treatment, the content of chlorophyll b in the STTM171 plants was significantly higher than that of the control. This may be related to the secondary regulatory effect of Se. How it is regulated still needs further experiments to analyze. Although the current TEM observations suggest abnormal chloroplast structures in STTM171 and MsSCL6-OE plants—such as membrane damage and partial disorganization of thylakoid layers—it is essential to conduct a more comprehensive quantitative analysis of these structural changes, including but not limited to expanding sample sizes and conducting rigorous morphometric assessments (Fig. 6). Moreover, the module in some plants has also been shown to affect various phenotypes, such as plant height, leaf structure, the number of lateral branches, and flowering time [36, 42, 43]. However, no corresponding phenotypic changes were obviously observed in alfalfa. It may be attributed to the dosage compensation action among different gene copies or other metabolic pathways in alfalfa, which is an autotetraploid plant. qRT-PCR data showed that *MsSCL6* expression was significantly downregulated in MsmiR171-OE plants. In contrast, the silencing of MsmiR171 relieved the inhibitory effect and significantly increased *MsSCL6* expression (Fig. 3C). These results showed that MsmiR171 represses *MsSCL6* to promote chlorophyll accumulation.

Chlorophyll molecules play a crucial role in capturing light energy and facilitating electron transfer in the process of photosynthesis [44]. MsmiR171 overexpression and *MsSCL6* silencing significantly increased Tr, Pn, Ci, and Gs in alfalfa leaves (Figs 3 and 5). Conversely, STTM171 and *MsSCL6-*OE plants showed the opposite results (Figs 3 and 4). Photosynthesis drives crop yield and quality. Enhanced photosynthetic rates typically elevate the accumulation of starch, soluble sugars, and soluble proteins in crops [45], thus improving their nutritional value. Significant increases in soluble sugars, soluble proteins, and starch contents were observed in both MsmiR171-OE and TRV: *MsSCL6* plants (Figs 3 and 5), while the contents of soluble protein and starch in STTM171 and *MsSCL6*-OE plants were significantly decreased (Figs 3 and 4). These findings showed that MsmiR171 promotes chlorophyll biosynthesis by repressing *MsSCL6*, thereby improving photosynthesis efficiency and promoting material accumulation in alfalfa.

Se can enhance photosynthesis and promote the accumulation of soluble proteins, sugars, and anthocyanins in radish sprouts, rice, and stevia [6–8]. In this study, Se application deepened leaf color, and respectively increased the content of soluble protein, soluble sugar, and starch, thereby improving photosynthesis in alfalfa plants (Figs 3–5). The expression levels of MsmiR171 and *MsSCL6* in different lines after Se application were detected. MsmiR171-OE plants exhibited increased MsmiR171 and decreased *MsSCL6* expression. Higher levels of *MsSCL6* transcripts were accumulated in STTM171 lines relative to the control with or without Se application. In *MsSCL6*-OE plants, *MsSCL6* transcript levels was significantly reduced after Se application, whereas in TRV: *MsSCL6* plants, *MsSCL6* expression remained at a relatively low level both before and after Se treatment, with no significant difference observed. This may be attributed to the gene silencing effect, which likely diminished its responsiveness to Se signals (Figs 3–5). Comprehensive analysis shows that Se application upregulates MsmiR171 expression, negatively regulates *MsSCL6*, and then affects chlorophyll biosynthesis. Although this study was conducted based on the previous findings and using a treatment condition of 100 mg L^−1^ and 12 h, there are still aspects that need to be further explored, such as whether the expression of MsmiR171 is time-dependent and the upstream regulatory factors responding to the Se signal.

The chlorophyll biosynthesis pathway must be precisely regulated through enzymatic control, as gene expression directly impacts chlorophyll content and photosynthetic capacity [46]. qRT-PCR revealed significant differential expression of *MsPOR* in overexpressed and silenced alfalfa plants (Figs S4 and S5). POR encodes protochlorophyllide oxidoreductase that catalyzes the limiting step conversion of protochlorophyllide to chlorophyllide in chlorophyll biosynthesis [47]. SlMYB72 and SlBEL11 regulate chlorophyll biosynthesis and chloroplast development by directly regulating *SlPORB* [46, 48]. In *Arabidopsis*, miR171 negatively regulates chlorophyll biosynthesis by targeting *SCL6*/*22*/*27* genes, which repress light-induced expression of *POR* [24]. Through yeast one-hybrid and dual-luciferase assays, it was found that MsSCL6 binds to the promoter of the *MsPOR* gene and inhibits its transcription activity (Fig. 7). It has been previously reported that SCL6 can bind to GT elements in *Arabidopsis* and cotton [23, 24]. By analyzing the cis-elements of the *MsPOR* gene promoter, we speculated that MsSCL6 may bind to the *MsPOR* gene promoter through the GT-box (Fig. 7F).

**Figure 8:**
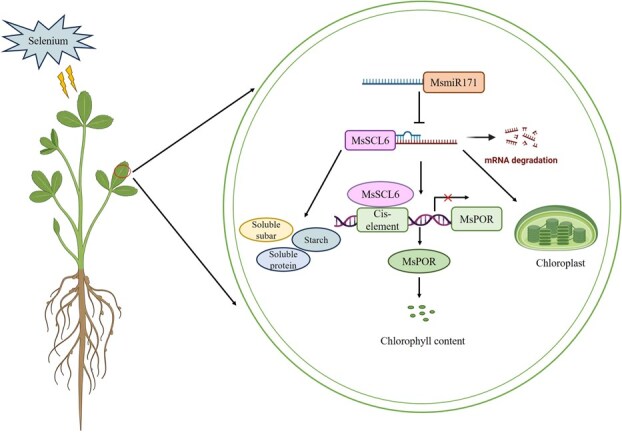
A schematic overview of the role of the MsmiR171-*MsSCL6* module in the regulation of chlorophyll biosynthesis in alfalfa. Under Se application, the expression level of MsmiR171 increased and negatively regulated the expression of the *MsSCL6* gene. MsSCL6 can inhibit the expression of the *MsPOR* gene by binding to unknown cis-elements and affect chlorophyll biosynthesis.

Based on these results, we propose a regulatory model in which Se induces MsmiR171 upregulation in alfalfa, which negatively regulates *MsSCL6* expression and thereby indirectly upregulates *MsPOR* expression to promote chlorophyll biosynthesis (Fig. 8). Here, the mechanism was first elucidated that the MsmiR171-*MsSCL6* module mediates Se-modulated chlorophyll biosynthesis in alfalfa. This research offers a theoretical foundation for developing high-quality forage that is rich in Se.

## Materials and methods

### Plant materials and treatments

Alfalfa (*M. sativa* L. cv. Kangsai), a high Se accumulation variety, was obtained from the team of Professor Chengzhang Wang at Henan Agricultural University. Alfalfa seeds were water-soaked, germinated, and transplanted into soil, then cultivated at 25°C under 16/8-h light/dark cycles with 70% humidity. The biofortification procedure employed sodium selenite [Na2SeO3, Se(IV)] at 0 and 100 mg L^−1^ concentrations, following Wang *et al.* [31]. After spraying Se for 12 h, leaf samples of different treatments were collected for RNA extraction and physiological index determination.

Transient expression was conducted in the three-leaf stage of *N. benthamiana* under standard conditions (25°C, 16 h/8 h light/dark).

### Bioinformatics analysis

psRNATarget predicted potential target genes of MsmiR171 in alfalfa (https://www.zhaolab.org/psRNATarget/) [49]. MsSCL6 sequences were aligned for evolutionary analysis, followed by construction of a neighbor-joining phylogenetic tree in MEGA 11 (1000 bootstraps) [50].

### Subcellular localization assay

The coding region of *MsSCL6* without termination codes was amplified and subcloned into the pCAMBIA-1300-35S-EGFP vector, so that *MsSCL6* was fused with EGFP at its N-terminus. The *MsSCL6*-EGFP construct was transiently expressed in *N. benthamiana* leaves [51]. The pCAMBIA-1300-35S-EGFP empty vector served as a control. Images of the samples were captured with a confocal microscope 48–72 h post-treatment.

### Gene expression analysis

Total RNA isolation was performed with an RNA Kit. For cDNA synthesis, follow the manufacturer’s instructions (Transgen, Beijing, China) with miR171-specific stem-loop primer and oligo (dT) primer. The Universal SYBR qPCR Mix (Qingke Bio) was used for qPCR analysis. To normalize the data, the *MsU6* and *MsActin* genes were employed. The 2^−ΔΔCt^ method was applied to calculate relative expression levels, with three biological replicates. The primers used for qRT-PCR assays are listed in Table S4.

### β-Glucuronidase histochemical staining and enzyme activity analysis

The GUS assay was used to identify the interaction between MsmiR171 and its target gene. First, the CDS of *MsSCL6* and *MsmSCL6* (with altered MsmiR171 target site but identical amino acid sequence) were cloned separately into the pBI121, generating the *MsSCL6*-GUS and *MsmSCL6*-GUS constructs. The MsmiR171 sequence was cloned into pBI121 by replacing the GUS gene, generating the pBI121-MsmiR171 construct. The pBI121-GFP vector was generated by replacing the *GUS* sequence in the pBI121 vector with the *GFP* sequence to serve as a negative control. Bacterial suspensions of pBI121, MsSCL6-GUS, wild-type Agrobacterium, MsmiR171, and MsmSCL6-GUS were individually adjusted to an OD600 of 0.6 in infiltration buffer. Subsequently, mix these cultures with the strain harboring the PBI121-GFP construct at a 1:1:1 volume ratio, ensuring that the final OD600 of the mixture is approximately 0.6. The resuspended bacterial solutions were injected at room temperature in the dark for 2–3 h before infiltration. Four injection sites per leaf were treated and stained according to Zhang *et al.* [52] and Jefferson *et al.* [53]. At 72 h postinfiltration, leaf samples were assayed for GUS activity using a GUS enzyme activity assay kit.

### Dual-luciferase assays

To verify the relationships between MsmiR171 and *MsSCL6*, and between MsSCL6 and *MsPOR* gene promoter, the MsmiR171 sequence and the CDS of *MsSCL6* were inserted separately into pGreen II 62-SK vector, and the *MsPOR* promoter, the CDS of *MsSCL6* and *MsmSCL6* were separately inserted into pGreen II 0800-LUC vector. The primer sequences used are listed in Table S4.

At 72 h postinfiltration, transformed leaves were analyzed by chemiluminescence imaging (Tanon 5200) [54]. LUC/REN values were measured with the Dual-Luciferase Reporter Assay Kit (E1910).

### Y1H assays

The CDS of *MsSCL6* was cloned into the pGADT7 vector, generating pGADT7-*MsSCL6* recombinant plasmids, while the promoter fragment of *MsPOR* was cloned into the pHIS2 vector to form the *MsPOR*-pHIS2 construct. The primer sequences used are listed in Table S4. Following cotransformed into Y187 yeast, then grown on SD-Trp/-His/-Leu + 3-AT media.

### Vector construction and plant transformation

The precursor sequence of MsmiR171 and the coding region of *MsSCL6* were independently cloned downstream of the *35S* promoter in the pBI121 vector, thereby constructing two overexpression vectors, MsmiR171-OE and *MsSCL6*-OE.

The STTM (short tandem target mimic) design for MsmiR171 was created following the methods outlined in previous studies [55], aiming to inhibit the activity of MsmiR171 by competing for its binding site. The construct included two incomplete copies of the MsmiR171 binding sites, connected by a 48-nt spacer. Each binding site region had a central three-nucleotide bulge. The fragment was then constructed into the pBI121 vector, named STTM171 (Fig. S3).

VIGS vector system for silencing *MsSCL6* based on TRV (Tobacco rattle virus) was conducted following the method outlined by Liu *et al.* [56], with a 346 bp fragment of *MsSCL6* CDS cloned into the pTRV2 vector, generating the TRV: *MsSCL6*. TRV:00 is the empty vector control used in this experiment, consisting only of the complete structure of the TRV virus (TRV1 and TRV2 empty plasmids, without the target gene insertion fragments). The constructs were delivered into alfalfa leaves using *Agrobacterium*-mediated transformation, following established methods [57]. The primer sequences used are listed in Table S4.

### Determination of photosynthetic pigment, soluble protein, sugar, and starch content

Pigment contents were extracted according to the method of Li *et al.* [58].

Soluble protein was quantified as the established method previously [59].

Soluble sugars were extracted as described by Masuko *et al.* [60].

Starch content was measured using a starch content determination kit (Sangon Biotech), with the absorbance of starch read at 620 nm and the starch content expressed as mg g^−1^.

### Determination of photosynthetic index

Alfalfa leaves with uniform growth and light were randomly selected, and three plants were taken from each treatment. The following photosynthetic parameters were measured using a Li-6800 portable photosynthesis analyzer (Li-COR, USA) between 9:00 and 11:30 a.m.: net photosynthetic rate (Pn), stomatal conductance (Gs), intercellular CO_2_ concentration (Ci), and transpiration rate (Tr). The measurements were taken under conditions of 1000 μmol·m^−2^·s^−1^ light intensity, a temperature of 25°C, and a CO_2_ concentration of 400 μmol·mol^−1^.

### The ultrastructure observation of leaves

The chloroplast structure of alfalfa leaves was observed by transmission electron microscopy, leaf treatment as described by Hu *et al.* [61].

### Statistical analysis

Statistically significant differences were analyzed using one-way ANOVA (*P* < 0.05) and Student’s *t*-test (^*^P < 0.05 or ^**^P < 0.01) with the SPSS 22.0 software. The data were expressed as mean ± SD and plotted using GraphPad Prism 9 software.

## Supplementary Material

Web_Material_uhaf305

## Data Availability

All data supporting this study are included in the article and its supplementary materials.
