# Supplementary material for: MsmiR171 targets MsSCL6 to mediate selenium-regulated chlorophyll biosynthesis in alfalfa
Source: Hortic Res. 2025 Nov 13;13(2):uhaf305. doi: 10.1093/hr/uhaf305 (PMC12936443; doi:10.1093/hr/uhaf305)
Supplement: Web_Material_uhaf305 [file web_material_uhaf305.zip › Supplementary information.docx]

**Supporting Figures**


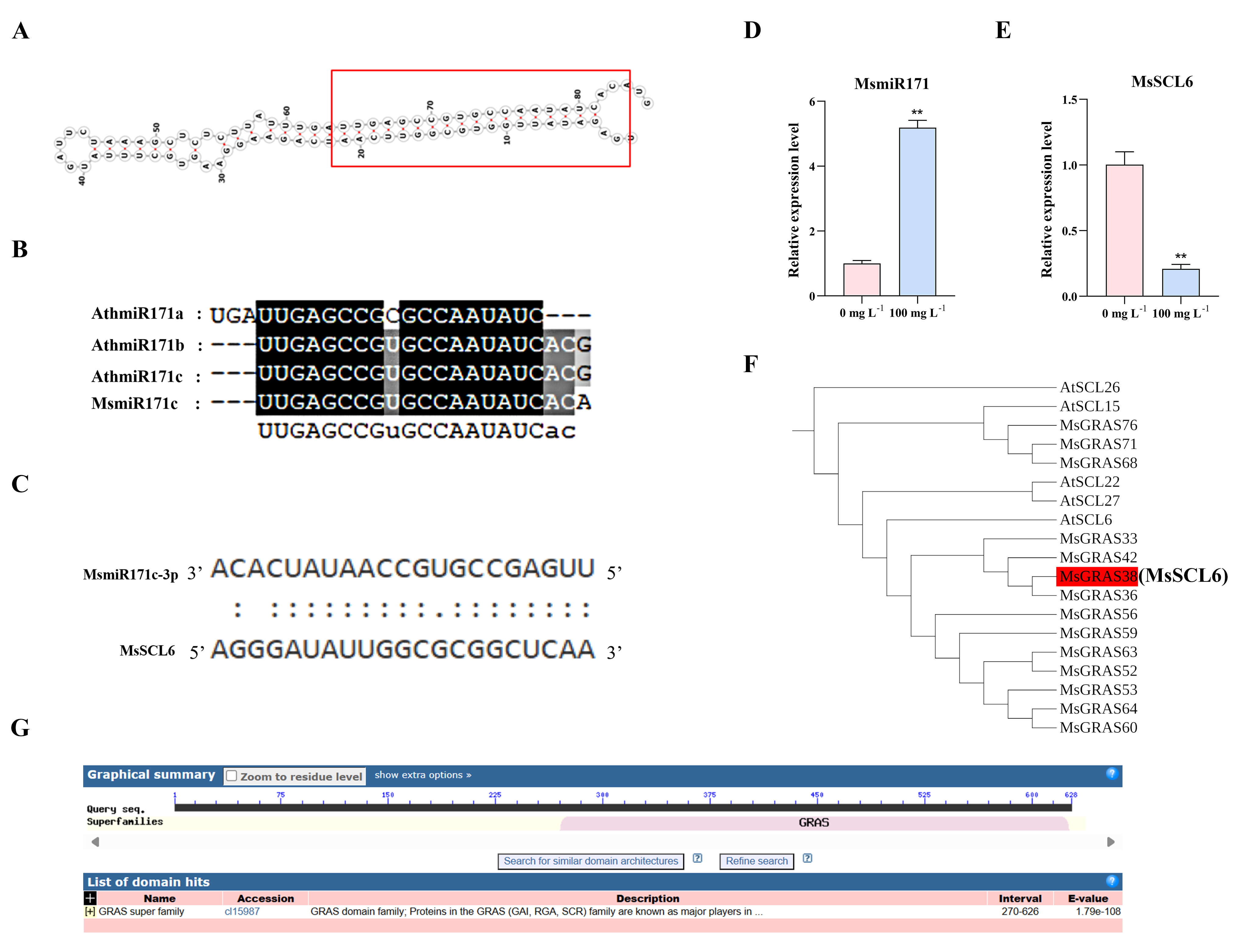


**Figure S1**. Identification of alfalfa MsmiR171 and *MsSCL6*. **A** Stem-loop structure of the MsmiR171 precursor. A red box represents the mature sequence. **B** Alignment of the mature sequences of alfalfa MsmiR171c and *Arabidopsis* miR171s using the ClustalW tool. **C** Prediction of MsmiR171 target gene by psRNATarget. **D** qRT-PCR verification of MsmiR171 after selenium application. Data were normalized according to the *MsU6* gene. **E** qRT-PCR verification of *MsSCL6* after selenium application. Data were normalized according to the *MsActin* gene. **F** Phylogenetic tree analyses of MsSCL6 homologs. **G** The conserved domains of MsSCL6. The data is shown as mean ± SD (n=3). Significant differences were determined using Student's *t*-test (**P* < 0.05 and ***P* < 0.01).


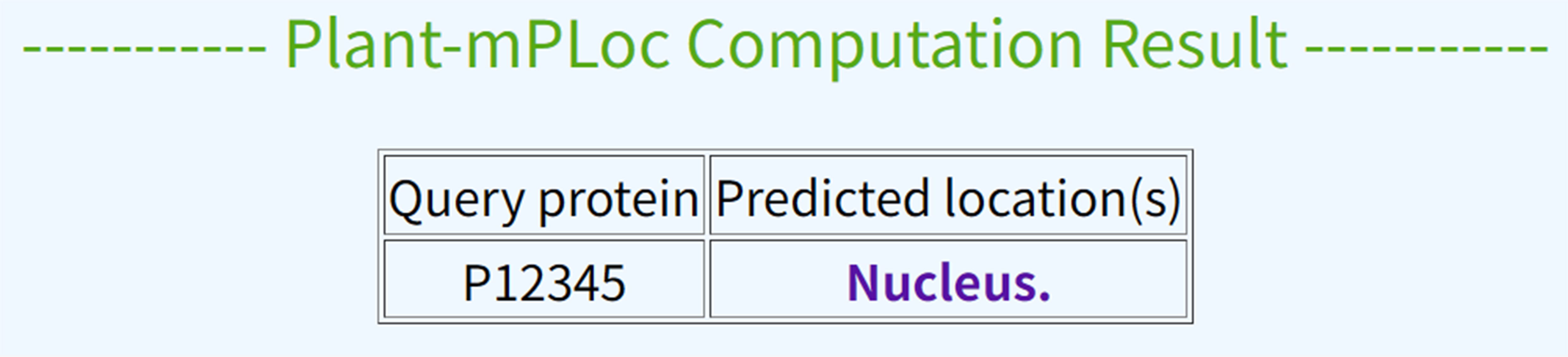


**Figure S2.** Subcellular localization prediction of target gene *MsSCL6*.

*
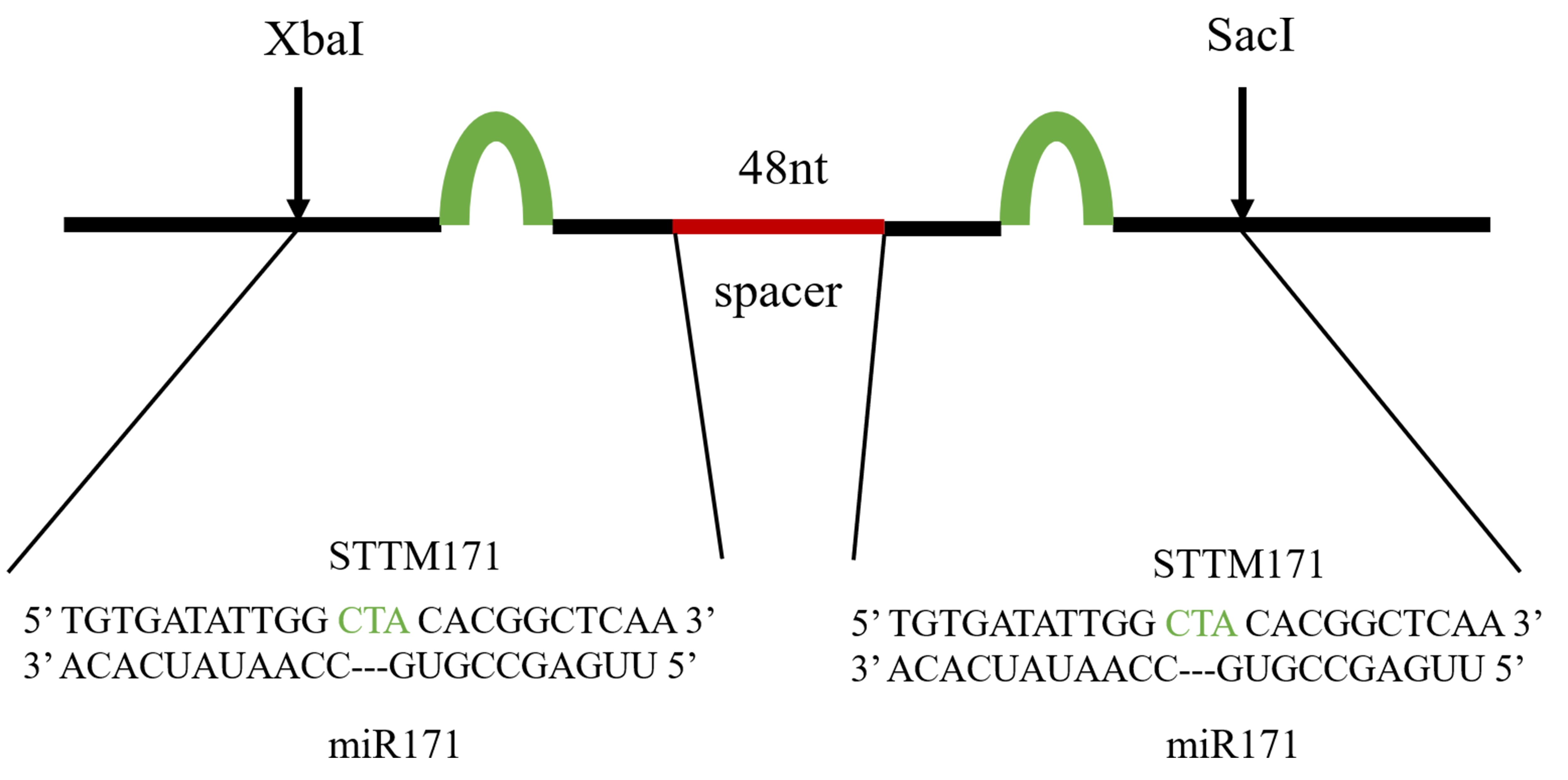
*

**Figure S3.** Construction diagram of STTM171.


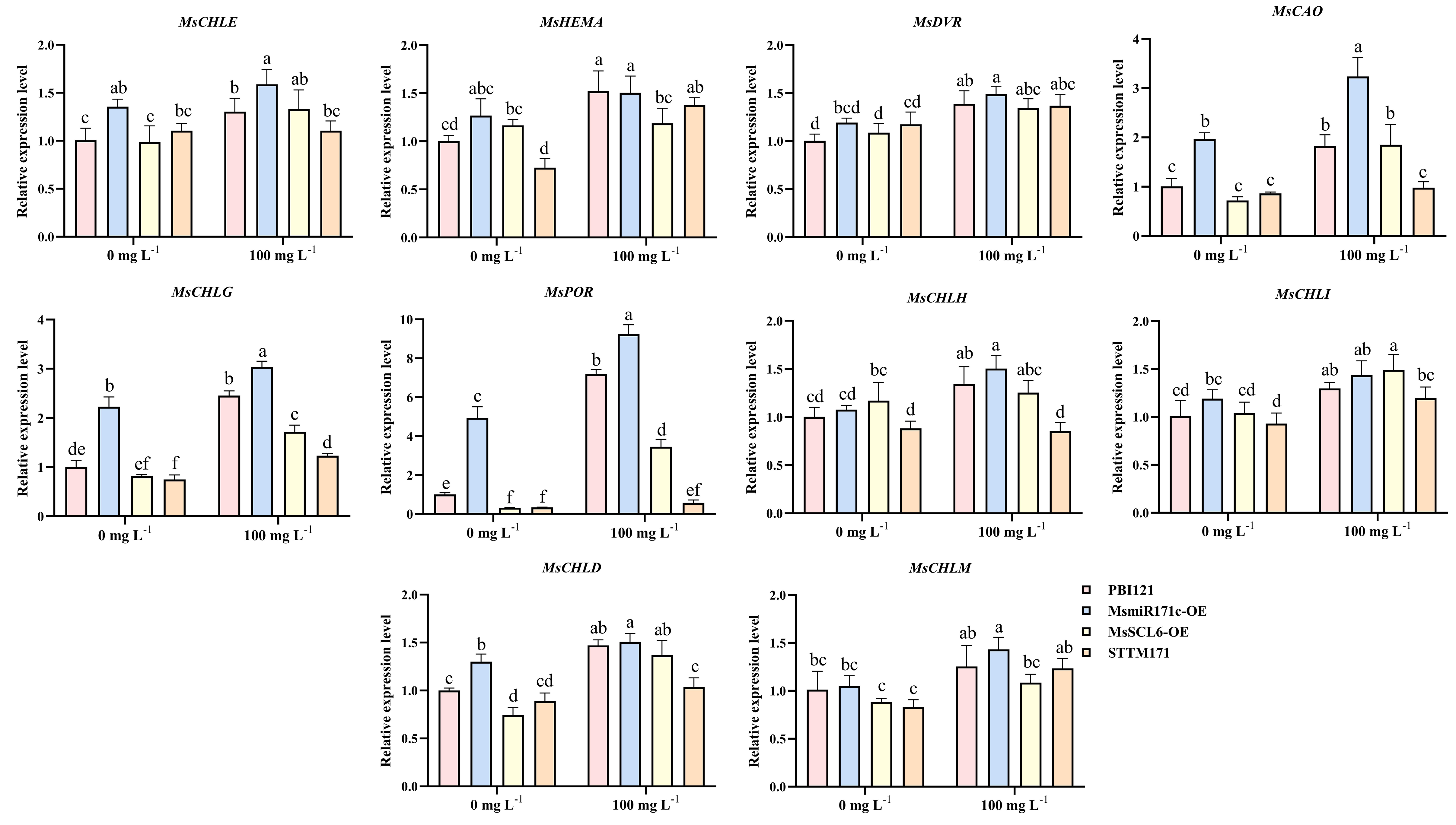


**Figure S4.** The effects of selenium application on the expression of chlorophyll synthesis-related genes in control, MsmiR171-OE, *MsSCL6*-OE, and STTM171 plants, with pBI121 used as the control. Data are presented as mean ± SD (n=3), and different letters indicate statistically significant differences at *P* < 0.05.


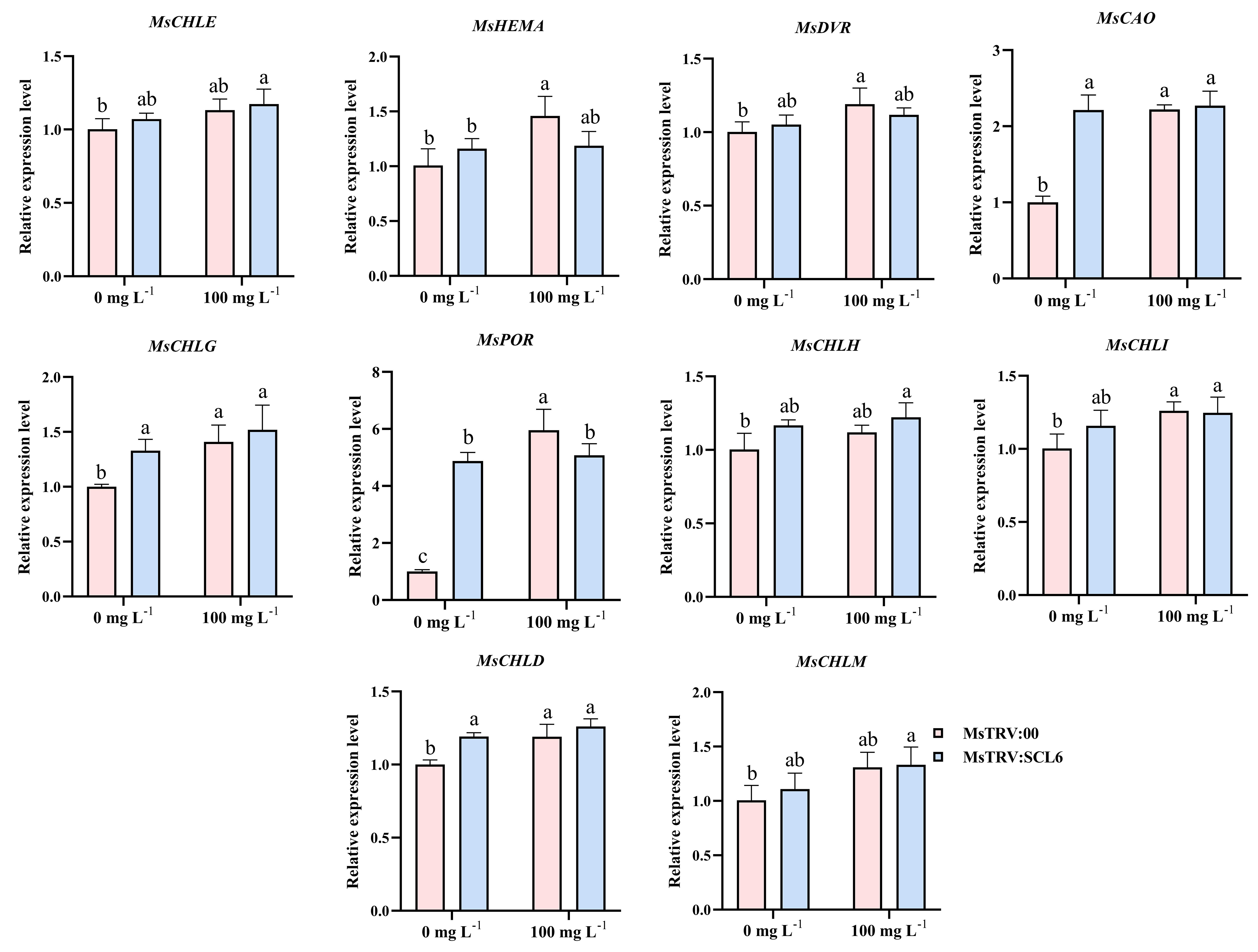


**Figure S5.** The effects of selenium application on the expression of chlorophyll synthesis-related genes in control and TRV: *MsSCL6* plants, with TRV: 00 used as the control. Data are presented as mean ± SD (n=3), and different letters indicate statistically significant differences at *P* < 0.05.
